# Patient experience after kidney transplant: a conceptual framework of treatment burden

**DOI:** 10.1186/s41687-019-0095-4

**Published:** 2019-01-30

**Authors:** Elizabeth C. Lorenz, Jason S. Egginton, Mark D. Stegall, Andrea L. Cheville, Raymond L. Heilman, Sumi Sukumaran Nair, Martin L. Mai, David T. Eton

**Affiliations:** 1 William J von Liebig Center for Transplantation and Clinical Regeneration, Mayo Clinic, 200 1st St. SW, Rochester, MN 55905 USA; 2 Center for the Science of Health Care Delivery, Mayo Clinic, Rochester, USA; 3 Mayo Clinic Arizona Transplant Center, Mayo Clinic, Phoenix, AZ USA; 4 Department of Transplantation, Mayo Clinic, Jacksonville, FL USA; 5 Division of Health Care Policy and Research, Department of Health Sciences Research, Mayo Clinic, Rochester, MN USA

**Keywords:** Burden of treatment kidney transplant

## Abstract

**Background:**

Kidney transplant recipients face a lifelong regimen of medications, health monitoring and medical appointments. This work involved in managing one’s health and its impact on well-being are referred to as treatment burden. Excessive treatment burden can adversely impact adherence and quality of life. The aim of this study was to develop a conceptual framework of treatment burden after kidney transplantation. Qualitative interviews were conducted with kidney transplant recipients (*n* = 27) from three Mayo Clinic transplant centers. A semi-structured interview guide originally developed in patients with chronic conditions and tailored to the context of kidney transplantation was utilized. Themes of treatment burden after kidney transplantation were confirmed in two focus groups (*n* = 16).

**Results:**

Analyses confirmed three main themes of treatment burden after kidney transplantation: 1) work patients must do to care for their health (e.g., attending medical appointments, taking medications), 2) challenges/stressors that exacerbate felt burden (e.g., financial concerns, health system obstacles) 3) impacts of burden (e.g., role/social activity limitations).

**Conclusions:**

Patients describe a significant amount of work involved in caring for their kidney transplants. This work is exacerbated by individual, interpersonal and system-related factors. The framework will be used as a foundation for a patient-reported measure of treatment burden to promote better care after kidney transplantation.

## Background

After kidney transplantation, patients face a life-long regimen of medications, lifestyle changes, self-care and medical appointments. Some construe of such tasks as the ‘work involved in being a patient’ [1]. Healthcare providers frequently contribute to this work and hold transplant recipients accountable for managing their health. The cumulative workload of treatment and self-management and its impact on patient well-being is referred to as treatment burden [2]. Treatment burden involves many aspects of care, including medication and monitoring burden, interpersonal conflict and economic challenges [3]. Treatment burden has been described in multiple medical conditions, including stroke, heart failure, diabetes, cystic fibrosis and cancer [4–7]. However, it is understudied in transplant patients.

Treatment burden can adversely affect patient outcomes. Patients who experience excessive treatment burden may not have the abilities or resources necessary to meet the demands of their health [8]. Patients who struggle to meet the demands of caring for their health may over- or underutilize healthcare, have poorer quality of life and demonstrate nonadherence to prescribed therapy [8, 9]. Treatment burden after kidney transplantation is a promising area of study because it is a potentially modifiable risk factor for adverse outcomes. Providers working with patients struggling with excess treatment burden could modify burdensome aspects of care, such as medication side effects and financial challenges. In addition, treatment burden could be incorporated into clinical practice guidelines to improve shared decision making between patients and providers [10]. Robust measurement of treatment burden could facilitate providers in their efforts to reduce it. As with any new measure, developing a patient-reported measure of treatment burden after kidney transplantation should involve both qualitative and quantitative research methods. First, direct input regarding treatment burden needs to be solicited from kidney transplant recipients using qualitative interviews and focus groups to derive a conceptual framework of treatment burden after kidney transplantation [2]. The conceptual framework describes the issues and domains of treatment burden to be represented as questions in the final measure. Using the framework as a foundation, a pilot measure is then drafted and validated using survey methods.

The goal of this study was to take the first steps toward developing a patient-reported measure of treatment burden after kidney transplantation by conducting qualitative interviews and focus groups with kidney transplant recipients and adapting a general framework of treatment burden developed in patients with multiple chronic conditions for use after kidney transplantation [2]. This general framework was chosen because many of the issues embedded within it are relevant to kidney transplant recipients (i.e. taking medications, monitoring health status, financial challenges, impact of treatment on life, etc.), and many kidney transplant recipients have diagnoses of multiple chronic conditions.

## Methods

### Qualitative interviews

A representative sample of adult kidney transplant recipients from the outpatient kidney transplant clinics at Mayo Clinic Rochester, Mayo Clinic Arizona and Mayo Clinic Florida was identified using a purposive sampling strategy. Patients were recruited via phone and interviews were conducted by EL (clinician), KS (qualitative analyst) or JE (qualitative analyst) via phone or in clinic from 9/2016 through 5/2017. Interviews were conducted using a semi-structured interview guide developed in prior studies involving patients with multiple chronic conditions [11] with some modifications to tailor it to the context of kidney transplantation. Patients were asked open-ended questions about their health, how they care for their medical conditions, impact of self-care on their lives, and stressors that affect their ability to care for their health. Demographic information and medical history were collected during the interviews and abstracted from the medical record and the Mayo Clinic transplant database. The majority of interviews lasted less than 60 min. Interviews were recorded and transcribed. Patients were not compensated for participating in the interviews. The qualitative interviews were approved by the Mayo Clinic Institutional Review Board. All patients provided oral informed consent and authorized the use and disclosure of their health information. Interviews were conducted until thematic content was saturated. Saturation was reached when further interviews revealed no new thematic content.

### Focus groups

After all but three interviews had been completed, two focus groups involving patients from Mayo Clinic Rochester were conducted in May 2017 to test the content validity of the conceptual framework developed from the interview data based on best practices outlined by Brod et al. [12] Eligible kidney transplant recipients from the outpatient transplant clinic at Mayo Clinic Rochester were recruited via phone. The focus groups were led by JE with EL present to take notes. The topic guide used during the focus groups was based on the conceptual framework treatment burden generated from the qualitative interviews with kidney transplant recipients. Feedback on the themes and subthemes of the conceptual framework and any unrepresented ideas were elicited. Patients who participated in the focus groups received a free meal and a parking pass. The focus groups were approved by the Mayo Clinic Institutional Review Board. All patients provided oral informed consent and authorized the use and disclosure of their health information.

### Data analysis

JE and EL independently reviewed and coded all interview transcripts by hand. DE adjudicated any disagreements in coding. After consensus was reached, the narrative data were then organized using NVivo software (Version 10; QSR International Pty Ltd., Melbourne, Australia). Framework analysis was used to identify key themes and subthemes as previously described [11]. Specifically, JE and EL developed a transplant-specific coding framework based on a framework of treatment burden developed in patients with multiple chronic conditions [2]. The framework was applied to code and index themes in subsequent interviews and was updated as additional interviews revealed new content. This process was continued until saturation was achieved and no new thematic content was identified.

Focus group data were used to evaluate the conceptual framework derived from the interviews and elicit any new issues not represented. Previous literature has recommended combining results of qualitative interviews and focus groups when constructing a conceptual framework for a patient-reported measure [12]. DE and EL analyzed the field notes and narrative transcripts from the two focus groups. A saturation grid outlining the themes discussed in each focus group was produced and a final conceptual framework of treatment burden following kidney transplantation was generated.

## Results

### Patient characteristics

During recruitment for the qualitative interviews, 31 patients were contacted, of whom 29 (93.5%) agreed to be interviewed. Of those 29 patients, 27 patients returned their Health Insurance and Portability and Accountability Act (HIPAA) forms and were included in the analysis. For the focus groups, 34 patients were contacted and 18 patients (52.9%) agreed to participate. Patients who declined participation frequently cited scheduling conflicts or a lack of time. Of these 18 patients, 16 patients attended one of two focus groups and were included in the analysis. Characteristics of the patients who participated in the qualitative interviews or focus groups are outlined in Table 1. Among the patients who participated in the qualitative interviews, the mean age was 56.0 years and the median time from transplantation was 24.0 months. The majority of the interviewees were male (59.3%), white/non-Hispanic (70.4%), finished some college/technical school (37.0%), married (55.6%) and retired (37.0%). Demographics of the focus group participants reflected demographics of Rochester, Minnesota and the surrounding area.

**Table 1:** Characteristics of study participants

Variable	Qualitative interviewees (*n* = 27)	Focus group participants (*n* = 16)
Age, years		
Median	56.0	50.5
Range	19.0 to 84.0	19.0 to 73.0
Gender		
Female	11 (42.3%)	9 (56.3)
Male	16 (59.3%)	7 (43.8)
Race/ethnicity		
White non-Hispanic	19 (70.4%)	13 (81.3%)
Black non-Hispanic	3 (11.1%)	1 (6.3%)
White Hispanic	4 (14.8%)	0 (0%)
Native American	1 (3.8%)	0 (0%)
Asian	0 (0%)	1 (6.3%)
Other	0 (0%)	1 (6.3%)
Time since transplant, months		
Median	24.0	81.0
Range	< 1 to 195	3–245
Donor type		
Living related	9 (33.3%)	5 (31.3%)
Living unrelated	7 (25.9%)	4 (25.0%)
Deceased	11 (40.7%)	7 (43.8%)
Cause of end-stage renal disease		
Glomerulonephritis	8 (29.6%)	7 (43.8%)
Diabetes	7 (25.9%)	1 (6.3%)
Polycystic kidney disease	5 (18.5%)	1 (6.3%)
Hypertension	1 (3.7%)	0 (0.0%)
Other	2 (7.4%)	7 (43.8%)
Unknown	4 (14.8%)	0 (0.0%)
History of prior kidney transplant	3 (11.1%)	3 (18.8%)
History of pre-transplant dialysis	17 (63.0%)	9 (56.3%)
History of cardiovascular disease	3 (11.5%)	2 (12.5%)
Diabetes mellitus	9 (34.6%)	5 (31.3%)
Mayo Clinic Transplant Center site		
Rochester	17 (63.0%)	16 (100.0%)
Arizona	5 (18.5%)	0 (0%)
Florida	5 (18.5%)	0 (0%)
Education		
High school graduate or less	7 (25.9%)	4 (25.0%)
Some college/technical degree	10 (37.0%)	3 (18.8%)
College graduate	4 (14.8%)	8 (50.0%)
Advanced degree	6 (22.2%)	1 (6.3%)
Marital status		
Never married	6 (22.2%)	4 (25.0%)
Married	15 (55.6%)	8 (50.0%)
Living with partner	3 (11.1%)	1 (6.3%)
Separated, divorced or widowed	3 (11.1%)	2 (12.5%)
Employment status		
Full-time employed	9 (33.3%)	4 (25.0%)
Part-time employed	3 (11.1%)	2 (12.5%)
Retired	10 (37.0%)	3 (18.8%)
Disabled or unemployed	5 (18.5%)	7 (43.8%)

### Interview results

**Fig. 1:**
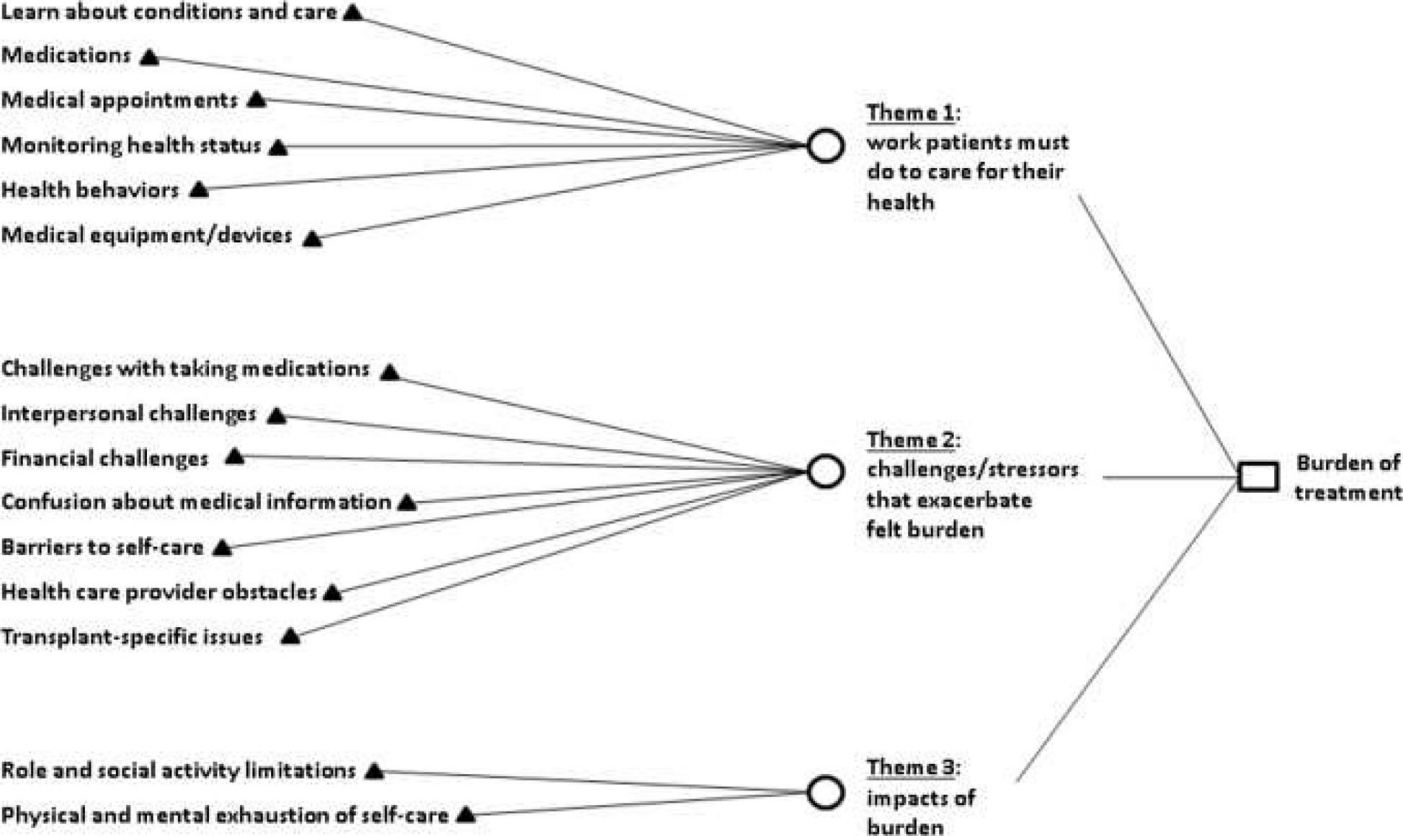
A conceptual measurement framework of treatment burden after kidney transplantation

Analysis of the transcripts from the qualitative interviews revealed the same three major themes identified in the prior study of treatment burden in patients with multiple chronic medical conditions [2]. Themes were identified by identifying patterns of meaningful content in patient responses. The three themes included the following: 1) work that patients must do to care for their health, 2) challenges/stressors that exacerbate felt burden and 3) impacts of burden. In addition, a new transplant-specific subtheme within Theme 2 was identified (see Fig. 1). These themes and subthemes are further explained below and illustrated using quotes from the qualitative interviews.

### Theme 1: Work patients must do to care for their health

All patients described work they must do to care for their health after kidney transplantation. The first subtheme of this work included *learning about their kidney transplant and how to care for it*:

I did go online to see what is a transplant, and that would show YouTube type of stuff. [45-year-old Caucasian male]

…in the hospital I bet it was every day they went through your medication, and then they would kind of give you like a test… [45-year-old Caucasian male]

Patients also described work involved in *taking medications*, including immunosuppressive medications, after kidney transplantation. Specifically, they often described taking large numbers of pills and needing to organize them and remember to refill them:

I take a lot of pills in the morning and then I take two more in the afternoon and then I take a bunch more at nighttime…I have so many damn pills I can’t remember. [64-year-old Caucasian male]

Once a week whenever I run out, and I think Saturday is when I run out, so Saturday night I just sit down and I have one of those little medical tray organizers, morning and night, and I’ll just start putting them in there based on what I take…[it takes] about an hour. [71-year-old Caucasian male]

There are some times that I wait too late to get my refills and I’ll go a day or two without my meds, but usually now what they did is they tell me to call them a week in advance, when I’m getting down. So what I did is I’ve got little stickers, and I put them on the calendar, and it’s a little medicine sticker that reminds me that this is the week you’ve got to call in your meds. [46-year-old Hispanic male]

The patients reported work involved in *attending medical appointments*, often with medical providers from multiple disciplines:

I have a dietician, an endocrinologist, a chiropractor, a general care doctor, a rheumatologist. [38-year-old Caucasian female]

It was like two days that we were going to be coming up for the [renal allograft] biopsy and then for the [transplant nephrology] consultation the second day…I always give a full day of travel. We travel on Sunday, biopsy Monday, consultation Tuesday, two days free, and then that…Friday for anything else if we had to change it. [70-year-old African American male]

You know, I love coming down [to the transplant center] because it saved my life, but now it’s getting to the point of everybody here saying everything’s okay. All of these tests and stuff like that, so it’s getting to a point where, ‘do I have to keep on coming down?’ I like coming down, but do I have to come down to hear them say everything’s okay? [68-year-old Native American male]

Some patients commented on work involved in *monitoring their health status*. Specifically, patients described challenges obtaining recommended blood draws locally and the need to monitor and log their vital signs, especially in the early post-transplant period, in record books provided across Mayo sites:

…the problem with [the clinical lab] is they take four days or five days to have the results of the blood…For me, as the patient, it is too late. If I have some parameters outside of range that could put my life at risk…sometimes they forget to do the tacrolimus. I would identify it, because basically I maintained a photocopy. I highlight the photocopy and what are the labs that they have to do. Every time I go there I say, ‘Please, mark this one, this one, this one.’ [48-year-old Hispanic male]

…we have a book that you get as part of the transplant, a three-ring binder, and it has some forms in it. When we use them up, we just go there and get some new ones. It has you track your medication and your vitals. We have a pretty good routine where we just sort of keep it all in a special little set of drawers I got for them, and each morning I do weight, blood pressure, temperature, and in the evening it is blood pressure and temperature. [67-year-old Caucasian female]

Patients described *health behaviors* after kidney transplantation that require work, including staying hydrated and avoiding certain foods:

I try to stay hydrated because I know it’s great for the kidney. [31-year-old Caucasian female]

Of course being mindful of your food…what things to avoid…food safety as well as those things that I can never eat again, such as the pomegranate and grapefruit. [67-year-old African American female]

Interviewees noted work associated with *post-transplant medical equipment/devices*, including bladder catheters, intravenous catheters and arteriovenous fistulas:

I’m here because of recurring bladder infections…Right now I have a [bladder catheter]. [81-year-old Caucasian female]

I did originally have to be placed back in the hospital, and I was given antibiotics through [an intravenous catheter] and also I had a [peripherally inserted central catheter] in...My son was a big help…he was kind of familiar with putting [intravenous catheters] in and things, being an [emergency medical technician] and fireman, so that kind of made me feel a little better. [67-year-old African American female]

So I’m thinking this [fistula] has something to do with me being just a little bit wobbly at times. [64-year-old Caucasian male]

### Theme 2: Challenges/stressors that exacerbate felt burden

In addition to theme 1, patients also described multiple issues that exacerbate their perceived treatment burden. For example, many patients described *challenges with taking medications*, including numerous side effects:

I had headaches which was one of the side effects. Then loss of hair, I had that. I had a slight undetermined tremor too, which has gotten worse, much worse… [67-year-old African American female]

What I am having a little bit of a problem with is the steroid. I know what the steroids are. I thought they were for getting pumped up, but they’re more like getting plumped up…I was 195 [pounds] one day, and now I’m like 204 or 209 [pounds]. [41-year-old Caucasian male]

Patients also described *interpersonal challenges with family and friends*:

She doesn’t know how my body feels. I understand she’s just trying to…help, but it’s just to the point where it’s hard to try and explain it to her, so a lot of times I don’t listen to her. I just go to bed and just cover my head up. [64-year-old Caucasian male]

I don’t like to talk about it because I like to keep some things private. Furthermore, growing up when I first got sick everybody knew it, everybody. I am kind of getting to where I’m older and I don’t really want people to know me as the sick girl or the girl with the kidney thing. I kind of want to be known as this really great person who is going back to school, but it’s like, “Oh, you have something wrong with you?” Yep, that was me. [31-year-old Caucasian female]

*Financial challenges* after kidney transplantation were frequently discussed. Specifically, patients described costs associated with traveling to their transplant center and difficulties with insurance coverage:

There are times when we come up here and it more or less takes grocery money away from us. I hate to say this, but she ends up going to the food shelf. [64-year-old Caucasian male]

I did find out that the shuttle there on the [transplant center] campus will pick you up anywhere on the campus and take you anywhere you want to go. So now I just call security and have the shuttle come and get me and save myself $5 a day or $15 a week, which adds up. [71-year-old Caucasian male]

[My insurance company] is not covering this why not, let’s dig in deep. As a patient I have had things where they have turned down and we are like, we are going to cover this. I like dig into it further and I call my insurance and I am like I want you to tell me why this wasn’t covered. ‘Oh, it was coded wrong.’ [31-year-old Caucasian female]

Patients commented on *confusion about medical information* provided after kidney transplantation, including not knowing which provider/medical center to contact with health concerns and distrust of the local facilities:

When I get sick I don’t know where I really should go [74-year-old Caucasian female]

I just don’t trust the [local lab] studies and that type of thing all that much. That’s why we started doing kits and sending the kits [to the transplant center] for the…[lab] studies. They’ll still do them at home, but we’ll compare them usually. [50-year-old Caucasian male]

Interviewees described *barriers to self-care* after kidney transplantation, including frequent urination from attempts to stay hydrated and musculoskeletal pain impairing their ability to exercise:

I wake up like 15 times a night. Sometimes I have to pee, because you know they have you drinking a lot of water, so sometimes at night I’m peeing it all out. [67-year-old Caucasian female]

Now my ankle is starting to bother me and I had surgery on that…now it hurts to walk…[64-year-old Caucasian male]

*Health care provider obstacles*, both related to the individual provider and system issues, were brought forward by patients during the qualitative interviews. Across Mayo Clinic sites, patients interface with transplant nurse coordinators, in addition to nephrologists, and are encouraged to establish care with local providers. Health care provider obstacles raised by patients included the following:

Well, it’s just like a few months ago I couldn’t afford to take my rejection medicine and I told my doctor at home. It seemed like she didn’t really do her job to make sure [the transplant center] knew, and then made it sound like it was my fault. [56-year-old Caucasian male]

All of our nephrologists…are not taking new patients. And the one I was seeing previously, he moved onto another clinic and is no longer with the hospital here…[38-year-old Caucasian female]

My biggest concern is I have a great nurse coordinator to do anything for me and get messages to people and do stuff, but it’s always a slow process, two or three days. It doesn’t happen quickly. It’s not her fault, it’s just the way the system is. The second thing is, there’s really no way to get in touch with your nephrologist there or any of the nephrologists that you have seen, except through the nurse coordinator. If you say to have them give me a call, then they don’t ever give me a call, they give a message to my coordinator and then she calls and tells me what they said. So I never have an opportunity, except when I go [to an] appointment, to talk to the nephrologist. [71-year-old Caucasian male]

It would be good if there was more communication between the internal medicine doctor and the doctors here so that they could kind of have a conference all or whatever to discuss, because it feels like he’s there and they’re here…there’s no real discussion. [62-year-old Caucasian male]

A new subtheme of Theme 2 identified in the interviews included *transplant-specific challenges*. These challenges mainly involved conflicts with the organ donor and fear of transplant failure:

It was actually uttered, ‘…and I gave you one of my kidneys.’ First of all, I never told you that I wasn’t thankful that you gave me one of your kidneys…It’s done. It’s in the past. It’s not that I’m not grateful, but you can’t throw that at me. It was a choice you made. I didn’t ask you to make it. I never asked you to make it, so that never needs to be uttered again. [39-year-old Caucasian male]

The top of the list is always the kidney. I want it to go as long as it possibly can. I know somewhere down the road I will have another transplant, but I try not to just sit and be like I’m getting a transplant someday. I don’t want to put that into my mind because then I will get nervous and I’ll be like, ‘Okay, I have to plan to do this first and I have to get this done.’ [31-year-old Caucasian female]

Well, you’re always going to worry. You were sick once, and you’re afraid you’re gonna get sick again. You are gonna be worried about it a lot. When you get that sick, you don’t wanna get that sick again. [19-year-old Caucasian female]

### Theme 3: Impacts of burden

Lastly, patients described how the burden of treatment impacted their life. Specifically, they described how their burden created *role and social activity limitations*:

I don’t do a lot of walking…I am lacking energy. And I used to be a goer. [74-year-old Caucasian female]

…they don’t want you getting sick, so they kind of tell you to stay away from bigger crowds, because your immune system isn’t that great… You can’t even go out to buffets; they don’t want you eating there. As well as your friend is like, ‘Can you come out and do…’ No, you can’t, because you just don’t want to get sick. Sometimes they don’t understand why, but you have to say, ‘No, I’m sorry, I can’t do that stuff.’ [64-year-old Caucasian male]

They also described *physical and mental exhaustion of self-care*:

I feel overwhelmed. Absolutely overwhelmed. [65-year-old African American female]

I am constantly examining what I can do better. [41-year-old Caucasian male]

…everything has to be dealt with, you just gotta remember sometimes you gotta take baby steps. If you try to do it all at one time and you know deal with the whole basket…then you get overwhelmed and they you get lost and sometimes you need to back down, back off…[51-year-old Caucasian male]

### Focus group results

Focus groups (*n* = 16) were used to confirm the themes and subthemes that emerged during the qualitative interviews. The majority of the interview themes and subthemes that emerged during the qualitative interviews also arose during the focus group discussions (see Table 2). No new themes emerged during the focus groups demonstrating that content saturation and content validity was achieved. The prior conceptual framework of treatment burden in patients with multiple chronic medical conditions [2] was revised to include a transplant-specific subtheme within Theme 2 (challenges/stressors that exacerbate felt burden). The conceptual framework of treatment burden after kidney transplantation appears in Fig. 1.

**Table 2:** Saturation grid of themes and subthemes represented in qualitative interviews and focus groups

Themes and subthemes	Frequency of endorsement (%)		
	Qualitative interviews (*n* = 27)	Focus group 1 (*n* = 7)	Focus group 2 (*n* = 9)
Theme 1: work patients must do to care for their health			
--Learn about conditions and care	59	100	100
--Taking medications	82	100	100
--Medical appointments	100	100	100
--Monitoring health	52	100	100
--Health behaviors	89	100	100
--Medical equipment/devices	26	100	100
Theme 2: challenges/stressors that exacerbate felt burden		100	100
--Challenges with taking medication	100	100	100
--Interpersonal challenges	30	100	100
--Financial challenges	85	100	100
--Confusion about medical information	59	100	100
--Barriers to self-care	41	100	100
--Health care provider obstacles (individual provider)	41	100	100
--Health care provider obstacles (system issues)	67	100	100
--Transplant-specific challenges	100	100	100
Theme 3: impacts of burden		100	100
--Role/social activity limitations	89	100	100
--Physical/mental exhaustion of self-care	52	100	100

## Discussion

We have developed a conceptual framework of treatment burden after kidney transplantation using qualitative interviews and focus groups involving kidney transplant recipients from multiple Mayo Clinic transplant centers. This conceptual framework illustrates the domains of treatment burden after kidney transplantation which will be used as foundation for a new patient-reported measure. Similar to a prior framework of treatment burden in patients with multiple medical conditions [2], our framework identified three major themes of treatment burden after kidney transplantation: 1) work patients must do to care for their health, 2) challenges/stressors that exacerbate felt burden and 3) impacts of burden. However, in addition, we identified a kidney transplant-specific subtheme reflective of condition-specific issues that may exacerbate felt burden. Transplant-specific issues of treatment burden mainly involved conflicts with the organ donor and fear of renal allograft failure.

Although no formal studies of treatment burden have been conducted in kidney transplant recipients, prior qualitative research has revealed some insight into the challenges these recipients face. After kidney transplantation, patients have reported difficulty with medication side effects, declining health, fear of return to dialysis, overmedicalization of life, cost of immunosuppressive therapy and post-transplant complications such as malignancy, infection and cardiovascular events [13–17]. Our work expands on these previous qualitative studies and provides the direct patient input and conceptual framework needed to create a patient-reported measure of treatment burden after kidney transplantation. Although measures of treatment burden exist for various chronic conditions including chronic kidney disease, none have been validated in kidney transplant recipients [3, 9].

Developing and adapting measures of treatment burden for use after kidney transplantation has potential impact on both clinical practice and research. First, measuring treatment burden in practice will allow providers to identify transplant recipients struggling with excessive treatment burden. These patients may not have the resources to handle the work involved in taking care of their health and may be at high-risk of nonadherence to prescribed therapy. Second, assessing treatment burden in practice could help providers modify the treatment plan to lessen treatment burden and improve patient quality of life and adherence. For example, a patient struggling with side effects from a particular immunosuppressive medication could be transitioned to an alternate agent. A patient struggling with economic burden could be referred to social work for assistance with insurance coverage. We acknowledge that incorporating measures of treatment burden into clinical practice will likely require multiple steps, including integration of the tool into the medical record, provider education, and development of clinical action plans [18, 19]. Lastly, a patient-reported measure of treatment burden could also serve as an exploratory endpoint in clinical trials involving transplant recipients. Trial endpoints have historically centered on patient and allograft survival when in fact they can also include how a patient feels or functions [20, 21]. Given that long-term outcomes after kidney transplantation are not improving over time [22], researchers need to develop new therapies to which patients can adhere to long-term and not adversely affect their quality of life [23].

Our study has several limitations. Patients involved in the study were all transplanted at the Mayo Clinic which may limit generalizability of our findings. However, we conducted qualitative interviews with patients from Mayo Clinic Rochester, Arizona and Florida to increase geographic, socioeconomic and racial/ethnic diversity. For example, Mayo Clinic Rochester has a higher proportion of Caucasian recipients and living donor kidney transplantation compared to the other two Mayo sites. Another limitation of our study is that it may be difficult to generalize findings obtained from interviews and focus groups involving a relatively small sample of patients. However, our findings are an extrapolation and extension of prior studies involving patients with multiple chronic conditions [2, 11]. With the exception of transplant-specific issues, our findings are consistent with the results of these prior studies [2, 11]. Furthermore, we demonstrated content saturation in this cohort. Another study limitation which may have influenced results is our use of a semi-structured interview guide developed in a prior study of patients with multiple chronic conditions [11]. We chose to modify the existing guide rather than create a new one because many of the treatment burden issues of persons with multiple chronic conditions are germane to transplant recipients and most candidates for transplantation are coping with multiple, chronic conditions [24]. Consistent with best practice, we modified the original guide for later interviews as we learned more about the patient experience from the early interviews. A final limitation of our study is that patients who declined participation due to lack of time may have been experiencing high burden. This is an inherent difficulty with studies examining treatment burden. To counteract this, we attempted to recruit patients with a history of nonadherence or post-transplant complications.

## Conclusions

We present the first study of treatment burden after kidney transplantation. Themes of treatment burden in kidney transplant patients are very similar to themes of treatment burden in patients with multiple chronic conditions, with the exception of additional burden from conflict with donors and fear of allograft failure. Our framework is currently being used to adapt a multidimensional, patient-reported measure of treatment burden for use after kidney transplantation [9]. A transplant-specific measure of treatment burden may promote identification of patients at high-risk for nonadherence and graft loss, allow for modification of treatment regimens and ultimately result in alleviation of treatment burden and improved patient-centered care. In addition, patient-reported measures like treatment burden could be used as exploratory endpoints in future clinical trials.

## Abbreviations

DE: David Eton
EL: Elizabeth Lorenz
HIPAA: Health Insurance and Portability and Accountability Act
JE: Jason Egginton
KS: Karen Schaepe

## References

[1] May C, Montori VM, Mair FS (2009). We need minimally disruptive medicine. BMJ.

[2] Eton DT, Ridgeway JL, Egginton JS (2015). Finalizing a measurement framework for the burden of treatment in complex patients with chronic conditions. Patient Relat Outcome Measures.

[3] Eton DT, Elraiyah TA, Yost KJ (2013). A systematic review of patient-reported measures of burden of treatment in three chronic diseases. Patient Relat Outcome Measures.

[4] Gallacher K, Jani B, Morrison D (2013). Qualitative systematic reviews of treatment burden in stroke, heart failure and diabetes - methodological challenges and solutions. BMC Medical Research Methodology.

[5] Gallacher K, Morrison D, Jani B (2013). Uncovering treatment burden as a key concept for stroke care: A systematic review of qualitative research. PLoS Medicine.

[6] Henry DH, Viswanathan HN, Elkin EP (2008). Symptoms and treatment burden associated with cancer treatment: Results from a cross-sectional national survey in the U.S. Support Care Cancer.

[7] Sawicki GS, Sellers DE, Robinson WM (2009). High treatment burden in adults with cystic fibrosis: Challenges to disease self-management. Journal of Cystic Fibrosis.

[8] May CR, Eton DT, Boehmer K (2014). Rethinking the patient: Using burden of treatment theory to understand the changing dynamics of illness. BMC Health Services Research.

[9] Eton DT, Yost KJ, Lai JS (2017). Development and validation of the patient experience with treatment and self-management (PETS): A patient-reported measure of treatment burden. Quality of Life Research.

[10] Dobler CC, Harb N, Maguire CA (2018). Treatment burden should be included in clinical practice guidelines. BMJ.

[11] Eton DT, Ramalho de Oliveira D, Egginton JS (2012). Building a measurement framework of burden of treatment in complex patients with chronic conditions: A qualitative study. Patient Relat Outcome Meas..

[12] Brod M, Tesler LE, Christensen TL (2009). Qualitative research and content validity: Developing best practices based on science and experience. Quality of Life Research.

[13] Jamieson NJ, Hanson CS, Josephson MA (2016). Motivations, challenges, and attitudes to self-management in kidney transplant recipients: A systematic review of qualitative studies. American Journal of Kidney Diseases.

[14] Pinter J, Hanson CS, Chapman JR (2017). Perspectives of older kidney transplant recipients on kidney transplantation. Clinical Journal of the American Society of Nephrology.

[15] Pinter J, Hanson CS, Craig JC (2016). 'I feel stronger and younger all the time'-perspectives of elderly kidney transplant recipients: Thematic synthesis of qualitative research. Nephrology, Dialysis, Transplantation.

[16] Afshar M, Rebollo-Mesa I, Murphy E, Murtagh FE, Mamode N (2012). Symptom burden and associated factors in renal transplant patients in the U.K. Journal of Pain and Symptom Management.

[17] Chamberlain, G., Baboolal, K., Bennett, H., et al. (2014). The economic burden of Posttransplant events in renal transplant recipients in Europe. *Transplantation*. 10.1097/01.TP.0000438205.04348.69 24732898

[18] Kroenke K, Talib TL, Stump TE (2018). Incorporating PROMIS symptom measures into primary care practice-a randomized clinical trial. Journal of General Internal Medicine.

[19] Santana MJ, Haverman L, Absolom K (2015). Training clinicians in how to use patient-reported outcome measures in routine clinical practice. Quality of Life Research.

[20] Sullivan EJ. Clincial Trial Endpoints. Presented at the: https://www.fda.gov/downloads/Training/ClinicalInvestigatorTrainingCourse/UCM337268.pdf. Accessed 21 Jan 2019.

[21] Stegall MD, Morris RE, Alloway RR, Mannon RB (2016). Developing new immunosuppression for the next generation of transplant recipients: The path forward. American Journal of Transplantation.

[22] Lamb KE, Lodhi S, Meier-Kriesche HU (2011). Long-term renal allograft survival in the United States: A critical reappraisal. American Journal of Transplantation.

[23] Stegall MD, Gaston RS, Cosio FG, Matas A (2015). Through a glass darkly: Seeking clarity in preventing late kidney transplant failure. Journal of American Society of Nephrology.

[24] Wu C, Evans I, Joseph R (2005). Comorbid conditions in kidney transplantation: Association with graft and patient survival. Journal of American Society of Nephrology.

